# Pathways From Family Violence to Adolescent Violence: Examining the Mediating Mechanisms

**DOI:** 10.3389/fpsyg.2021.611006

**Published:** 2021-02-09

**Authors:** Spencer D. Li, Ruoshan Xiong, Min Liang, Xiaohua Zhang, Wei Tang

**Affiliations:** ^1^Department of Sociology, Faculty of Social Sciences, University of Macau, Macao, China; ^2^Department of Social Work, College of Humanities and Social Sciences, Huazhong Agricultural University, Wuhan, China; ^3^Department of Sociology, Faculty of Social Sciences, University of Macau, Macao, China; ^4^Faculty of Law and Sociology, Nanning Normal University, Nanning, China; ^5^Social Work, Guangdong Pharmaceutical University, Guangzhou, China

**Keywords:** family violence, youth violence, intergenerational transmission, China, mediation

## Abstract

**Purpose:**

Past research has documented a significant relationship between family violence and adolescent violence. However, much is unknown about the processes through which this association occurs, especially in the non-Western cultural context. To address this gap, we propose an integrated model encompassing multiple pathways that connect family violence to adolescent violence. Specifically, this study investigates how family violence is related to adolescent violence through violent peer association, normative beliefs about violence, and negative emotions.

**Method:**

We tested the model using the two-wave survey data collected from a probability sample of more than 1,100 adolescents residing in one of the largest metropolitan areas in China in 2015 to 2016.

**Results and Conclusions:**

The results indicated that family violence predicted adolescent violence perpetration. Violent peer association, normative beliefs, and negative emotions, however, mediated much of the relationship between family violence and adolescent violence.

## Introduction

Family violence is a worldwide problem with a series of long-term adverse consequences for survivors, especially children, across their life span. Globally, the World Health Organization (WHO) has identified children as the most common victims of family violence (Timshel et al., 2017). According to child welfare data from Canada and the United States, corporal punishment and witnessing intimate partner violence (IPV) are the major forms of family violence inflicted on children (Canada, 2010; Smith et al., 2011; Renner and Whitney, 2012; Pecora et al., 2017). Prior research has shown that children who have been victims of child abuse or witnessed IPV are more likely to perpetrate violence later in their lives (Widom and Wilson, 2015; Renner and Boel-Studt, 2017).

Despite the evidence that family violence promotes adolescent violence, much is unknown about how the process takes place. Several studies have shown that violence experienced at home is positively related to psychological and behavioral problems that increase adolescent risk for violence perpetration. In this regard, the relationship between family violence and adolescent violence may involve multiple pathways (Liang et al., 2019; Liu et al., 2020; Perry et al., 2020). For instance, children exposed to family violence may have a higher tendency to accept violent norms that legitimatize the use of violence as a means to resolve personal and interpersonal problems (Xia et al., 2018). Additionally, adolescents raised in homes with higher levels of violence may develop more negative emotions and stronger association with violent peers, both of which are positively related to adolescent involvement in violent behavior (Ohbuchi and Kondo, 2015; Edelstein, 2018). There are strong theoretical reasons to expect that all of these factors—normative beliefs about violence, negative emotions, and violent peer association—play critical roles in linking family violence to adolescent violence. Studies conducted thus far have either ignored these important mechanisms altogether or explored only some of these processes. Further, to our knowledge, there has been no study conducted in China examining all these mechanisms when trying to understand the process of intergenerational transmission of violence, which has been a longstanding problem in the country but has received virtually no attention in empirical research (Chan, 2011; Widom and Wilson, 2015). By integrating these mechanisms into a theoretical model, the current study assesses the roles of normative beliefs about violence, negative emotions, and delinquent peer association in connecting family violence and adolescent violence using the two-wave survey data collected in China. In so doing, the study aims to provide a more comprehensive understanding of the relationship between the two forms of violence as they are manifested in Chinese society.

## Literature Review

### Family Violence and Adolescent Violence

Social learning theory maintains that delinquency including violent behavior is learned through interactions with intimate others, such as family members and peers (Akers, 1998). As applied to violence, social learning theory predicts that adolescents exposed to a high level of family violence are prone to violent behavior because they learn to act violently from their family members, especially parents. Children learn to perpetrate violence mainly through two learning processes: observational learning through emulating the violent behavior of role models such as parents and intergenerational transmission of attitudes that are conducive to violence (Sellers et al., 2005).

Prior research has provided strong support for social learning theory by demonstrating that experiencing physical abuse is positively associated with adolescents’ violent behavior (Duman and Margolin, 2007; Xia et al., 2018; Karsberg et al., 2019). For example, by analyzing the data collected from a national sample, Cuartas et al. (2020) found that physical punishment strongly predicted adolescents’ delinquent involvement, especially violent offending. The same conclusion was drawn by Greene et al. (2020) and Fenimore et al. (2019) in their systematic reviews of parenting practices and adolescent behavior problems. They found that children were more likely to manifest violent and aggressive behaviors if they experienced physical abuse in childhood. Similar results also emerged from the studies of Henry et al. (2001) and Henneberger et al. (2013), which showed that male and female adolescents were more likely to use violence in relationships if they had been subject to parental corporal punishment.

Apart from physical abuse, a growing body of empirical literature indicates that witnessing IPV is also associated with adolescent behavior problems, especially violence perpetration (Whitefield et al., 2003; Narayan et al., 2015). For instance, using two-wave longitudinal data collected from a representative sample of Chinese adolescents, Xia et al. (2018) found that children with higher exposure to IPV were more accepting of the use of violence and more likely to perpetrate violent acts than their counterparts who observed fewer violent incidents at home. In light of these findings, we posit the following hypothesis:

**H1:** Family violence is positively associated with adolescent violence.

### Family Violence, Normative Beliefs, and Adolescent Violence

The studies reviewed above point to the possibility that the psychological or behavioral characteristics of the adolescent act as mediators linking family violence to adolescent violence. One of the factors that has been cited in prior research is the adolescent’s normative beliefs about violence, which refer to a subjective appraisal of a specific violent act that is expected or desired under a given circumstance (Fishbein and Ajzen, 1980). According to social learning theory, youth develop a propensity for delinquency through learning the “values, orientations, and attitudes” that are favorable to committing delinquent acts from people close to them, including their parents (Akers and Jennings, 2009, p. 106). Consistent with this line of reasoning, adolescents who have more exposure to family violence would be more likely to develop normative beliefs that justify the use of violence as acceptable means to control and dominate others, which would in turn precipitate violent behavior.

Some studies have shown that experiencing violence at home increased individuals’ acceptance of violent norms, which in turn predicted future violent behavior. These patterns of relationships have been found in different adolescent demographic groups (Brendgen et al., 2002; Coohey et al., 2013) and across socioeconomic strata (Beyers et al., 2001). Markowitz (2001), for example, found that experiencing violence early in life increased favorable attitudes toward violence against others and violent attitudes positively predicted subsequent violence against both children and intimate partners. Viewing from the opposite direction, Mumford et al. (2016) found that youth grown up with positive parenting and high-quality parental relationship were less likely to become tolerant of violence and consequently were less prone to violent behavior in late adolescence and early adulthood. Based on these studies, we propose the following hypothesis.

**H2:** Normative beliefs about violence mediate the relationship between family violence and adolescent violence. Specifically, family violence will promote adolescents’ approve of the use of violence, thereby increasing their involvement in violent behavior.

### Family Violence, Negative Emotions, and Adolescent Violence

Exposure to family violence is also an established risk factor for negative emotions in adolescence (Baek et al., 2019; Caspi et al., 1994). Prior research has indicated that children who have experienced family violence are more likely to develop depression and anxiety, post-traumatic stress disorder, and a range of other emotional problems that are related to antisocial behavior (Johnson et al., 2002; Herrenkohl et al., 2005; Sternberg et al., 2006). The general strain theory proposed by Agnew (1992, 2013) provides a theoretical framework to understand the relationships among family violence, negative emotions, and adolescent violence. According to this theory, negative emotions operate as a leading cause of delinquent and violent behavior. External stressors such as family violence act as strains to increase adolescents’ negative emotions, which then lead to a higher likelihood of violence perpetration. Seen from this perspective, negative emotions should significantly mediate the relationship between family violence and adolescent violence.

Indeed, it has been well documented that negative emotions serve as a critical link between family violence and adolescent violence. A number of studies have shown that exposure to family violence increases negative emotions such as depression and anxiety in adolescence, and adolescents who harbor these negative emotions have higher levels of involvement in aggressive and violent behavior (Baek et al., 2019; Mrug and Windle, 2010; Kassis et al., 2013; Birkley and Eckhardt, 2015). For example, McCloskey and Lichter (2003) found that children grown up in families with high prevalence of marital violence became more depressed as adolescents and elevated depression mediated the impact of interparental violence on adolescents’ aggression.

Informed by the previous literature, we propose the following hypothesis.

**H3:** Negative emotions mediate the relationship between family violence and adolescent violence.

### Family Violence, Violent Peer Association, Violent Norms, Negative Emotions, and Adolescent Violence

Social learning theory (Akers, 1998) postulates that family violence increases juvenile delinquency and violence by strengthening adolescents’ association with violent peers. For adolescents exposed to family violence, deviant and violent peers might emerge as a vicarious family. When an adolescent is associated more with individuals who are involved in criminal and deviant behaviors or demonstrate pro-criminal attitudes, he or she is more likely to engage in the criminal/deviant behavior (Akers and Jennings, 2016; Hong et al., 2017).

Prior literature has shown that poor parenting, characterized by marital conflict and physical abuse, may foster children’s delinquent association (Xia et al., 2018; Manzoni and Schwarzenegger, 2019; Liu et al., 2020). As most juvenile delinquency happens in groups, adolescents’ affiliation with violent peers operates as a strong predictor of adolescents’ delinquent and criminal behavior, including violent behavior (Arriaga and Foshee, 2004; Dishion et al., 2010; Haggerty et al., 2013). Research has also shown that association with violent peers strengthens adolescents’ negative emotions such as depression and anxiety (La Greca and Harrison, 2005; Bacchini et al., 2011). Additionally, affiliation with violence-prone friends is related to a more positive attitude toward violence (Brendgen et al., 2002; Seddig, 2014). Based on this literature, we posit the following hypotheses.

**H4:** Violent peer association mediates the relationship between family violence and adolescent violence.
**H5:** Violent peer association increases negative emotions.
**H6:** Violent peer association promotes normative beliefs approving violence.

## The Current Study

**FIGURE 1:**
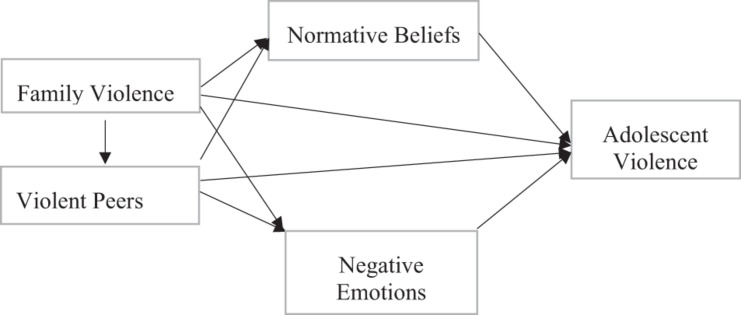
The theoretical model of the current study.

Although there are strong reasons to expect that normative beliefs about violence, negative emotions, and violent peer association all operate as important mechanisms linking family violence and adolescent violence, to our knowledge, no study has closely examined how all of these factors significantly contribute to the influence of family violence on adolescent violence using representative and longitudinal data. There are three reasons why it is important to examine all relevant pathways in an integrated model. First, it represents a fuller test of the social learning and general strain theories, which suggest that the influences of family violence on adolescent violence are effectuated through multiple mediating mechanisms involving normative beliefs, affective attributes, and peer relationships. Second, the omission of any important mediator in conceptualization and statistical analysis may run the risk of model misspecification, which can produce misleading research results. Third, from a policy perspective, the inability to understand all of the critical pathways to adolescent violence may undermine the effort to develop comprehensive strategies to prevent youth violence. Drawing on the social learning theory and general strain theory, this study integrates family violence, normative beliefs, negative emotions, violent peer association, and adolescent violence into a comprehensive model to assess the structural relationships among them using longitudinal data. The theoretical relationships among these concepts are illustrated in Figure 1. As depicted in Figure 1, family violence has a positive effect on adolescents’ violent peer association, normative beliefs approving violence, and negative emotions. Violent peer association is positively related to normative beliefs and negative emotions. Finally, all three mediators—violent peer association, normative beliefs approving violence, and negative emotions—are positively related to adolescent violence.

## Data and Measurement

### Data

The data used in the current study were from a two-wave longitudinal research project on family processes and delinquency. The institutional review board of the university that funded the project reviewed and approved the study design and procedures. We collected the data in one of the largest metropolitan areas. The research site had been a major city in China before the country opened up its economy to the world in the late 1970s, but it has developed into a highly populated and diverse regional urban center in recent years with mixed urban and suburban districts. It is now home to 30 million people, including millions of migrant workers and ethnic minorities.

To ensure the representativeness of the sample, we randomly selected eligible participants for the study using a three-stage stratified probability proportionate to size sampling procedure. In the first stage, we randomly selected three districts, including two urban districts and 1 suburban district. In the second stage, we randomly selected 1 suburban middle school, one urban middle school, one suburban high school, and one urban high school from each sampled district, resulting in a total of 12 schools. In the third stage, in each sampled school, we proportionately selected a random number of classes in the 7th, 8th, 10th, and 11th grades. Secondary schools in China, including middle schools and high schools, are 3 years in duration. Considering that 9th and 12th graders (the final years of middle and high school) would graduate before the start of the second wave of the survey, we did not include them in the survey.

We contacted the sampled schools to seek their support and cooperation for the study. If the sampled school or class refused to participate in this survey, we randomly selected a replacement school or class until the sample size was reached. Once we obtained the cooperation of a school, we visited the school to introduce our study and sample the students. We provided the schools with the written informed consent forms for both the students and their parents. The forms contained information about the background and objectives of the study, the survey procedures, and a summary of the questions about on the questionnaire. In addition, the consent forms clearly stated that the participation in this study was entirely voluntary, and the privacy and confidentiality of the respondents would be strictly protected. We also asked the students to provide contact information if they agreed to be followed up in the second wave. Only students who agreed to participate in both waves of the study and whose parents signed a consent form were included in the current study, which yielded 1,300 eligible participants. A paper-and-pencil survey was administered to the sampled students. Around the same month in the following year (2016), we conducted the second wave of survey in the same schools with the same class of students. As with many other school-based surveys of adolescents, such as NLSY (Benevides et al., 2020), we choose a 12-months interval between two waves since children may experience a significant change during their adolescence. It could be hard to capture these changes if the time gap was too long. The response rates for the Wave 1 and Wave 2 surveys were 97.20 and 96.73%, respectively. Additionally, 193 participants who had missing values on study variables, including the non-respondents, were excluded in the analyses, resulting in a final sample of 1,107.

### Variables and Measurement

In this study, the key variables were family violence, violent peer association, normative beliefs about violence, negative emotions, and adolescent violence. To the extent possible, standard instruments with demonstrated reliability and validity were used to measure these concepts. Most of the measurements comprise multiple indicators, which help improve reliability and reduce measurement errors. With the exception of family violence, all of the other key variables were measured using data collected in the second wave in 2016. During early and middle adolescences, adolescents’ beliefs, mental health, and peer network can undergo substantial changes over a relatively short period of time (Eccles et al., 1993; Chambers et al., 2003). For this reason, it is more appropriate to examine the contemporaneous effects using measures collected from the same year when assessing the interrelationships among normative beliefs, negative emotions, delinquent peer association, and violent behavior. Family violence, on the other hand, is more likely to have a lagged effect on adolescent violence through fostering a tendency for violent behavior by subjecting the adolescent to recurring exposure to violence at home. Therefore, it is more suitable to use wave-1 measures of family violence to predict wave-2 adolescent violence.

#### Adolescent Violence

Adolescent violence was measured by using a modified questionnaire from the Project on Human Development in Chicago Neighborhoods (PHDCN; Earls et al., 2006), which was administered in the survey in the second wave in 2016. The respondents reported the frequency of the following eight forms of violence they perpetrated during the last year: taking something from others with a threat, threatening someone with a knife or gun, hurting someone with a knife or gun, participating in a gang fight, hitting someone or threatening to hit someone, assaulting someone badly, robbing someone in a violent way, and having sex with someone against their will. All items were measured on a 7-point Likert scale, from 0 for “never” to 6 for “at least 1 time per day” (Anderson, 1997). It is a common practice in criminological research to use the sum of different delinquent acts to measure the level and type of delinquent involvement (Elliott and Huizinga, 1983; Li and Xia, 2018), especially when the original responses are assessed on an ordinal scale. We followed this approach by computing a total score as the measure of adolescent violence after dummy coding each of eight violent acts into 0 for “never” and 1 for all other answers.

#### Family Violence

Most studies conducted in this area have used measures of child-directed violence such as child physical abuse or child-witnessed violence such as interparental violence (Maxwell and Maxwell, 2003). To tap both dimensions of family violence, we included IPV and child physical abuse in our measure of the overall level of violence that the adolescent experienced in the home environment. The two forms of family violence have shown to be highly correlated in Chinese society (Chan, 2011). IPV was measured by the adolescents’ responses to four questions about how often their father (mother) hit their mother (father) and how often their father (mother) yelled at their mother (father). Physical abuse was also measured by four questions about how often the father (mother) beat the child for no reason and how often the father (mother) slapped/beat the child for doing something wrong. All of the questions had the following possible responses: “never,” “seldom,” “sometimes,” “often,” and “always.” A total score was computed by summing all of the items after they were converted to dichotomous measures with 0 representing “never” and 1 representing “seldom,” “sometimes,” “often,” and “always” (Kwong et al., 2003).

#### Violent Peer Association

Violent peer association was measured by two questions asking the respondents how many of their friends engaged in fighting, and in threatening and intimidating others. The answers ranged from 0 for “none” to 4 for “all of them.” We used the sum of the scores on the two items to measure violent peer association. The Cronbach’s alpha for the two items was 0.65.

#### Normative Beliefs Approving Violence

Normative beliefs were assessed using the general belief questions about aggression and violence developed by Huesmann et al. (1992), which consisted of five items. The questions included “in general, it is wrong to hit other people”; “in general, it is OK to yell at others and say bad things”; “In general, it is OK to take your anger out on others by using physical force”; “it is generally wrong to get into physical fights with others”; and “it is usually OK to push or shove other people around when you’re mad.” The answers ranged from 1 for “it’s really wrong” to 4 for “it’s perfectly OK.” We calculated the mean of the items after reverse coding some of the items so that the higher scores represented stronger approval of the use of aggression and violence. The Cronbach’s alpha for the eight items was 0.70.

#### Negative Emotions

The measure of negative emotions was selected from the Middle-School Student Mental Health Inventory (MMHI) developed by Wang et al. (1998) for Chinese students. The instrument consists of five subscales, including depression, anxiety, hostility, paranoid ideation, and interpersonal sensitivity and strain, and 30 questions in total (a full list of the questions is provided in Appendix 1). A factor analysis showed that the 30 items loaded onto the same factor representing negative emotionality. The answers for each question ranged from 1 for “never” to 5 for “always,” and the mean score was calculated, with a higher score indicating that respondents have more negative emotions. The Cronbach’s alpha for the 30 items was 0.96, and 0.82, 0.87, 0.84, 0.81, and 0.75 for the five subscales, respectively.

#### Age and Gender

Age and gender were included as the control variables in the current study. Age was measured by years ranging from 9 to 16 at wave 1 but its mean was nearly 14 years. Gender is a dichotomous variable with 0 for male and 1 for female. Each gender group accounted for about 50% of the sample.

### Data Analysis

We conducted two types of analysis: descriptive analysis and mediation effect analysis. The descriptive analysis described the attributes of the variables included in the regression models, while the mediation effect analysis tested the structural relationships among the key concepts by using a series of multiple regression models (Li, 2011). Depending on the distribution of dependent variables, we employed either negative binominal models or linear regression models to test the direct and indirect effects with Stata 14.2. Although the dataset contains a two-level structure with students nested within schools, we were unable to conduct a multilevel analysis because we did not collect data on the school level. School administrators were very reluctant to provide institutional data because of the concern that the information might be used to measure their job performance. A large number of them might have chosen not to participate in the survey had we insisted on collecting school-level data. As it stands now, the unit of analysis of our study remains at the individual level.

## Results

### Descriptive Analysis

Table 1 lists the sample’s characteristics, including age, gender, family violence, violent peer association, normative beliefs approving violence, negative emotions, and adolescent violence. For family violence, the average score was 3.45, indicating that the respondents typically experienced multiple forms of family violence. In terms of violent peer association, the average score was 0.64, indicating that the respondents had very few friends who were involved in violent behavior. As for normative beliefs about violence, the average score was 1.80, suggesting that the Chinese adolescents generally regarded the use of violence as “sort of OK.” The measure of negative emotions has a mean score of 2.07, indicating a moderate level of mental health problems in the sample as a whole. Finally, the average score of adolescent violence was 0.34, suggesting that the adolescents committed less than one-half of the eight types of violent behavior on average.

**TABLE 1:** Descriptive analysis of key variables (*N* = 1,107).

Variable	Mean	Std. Dev.	Min	Max
Age (years)	13.81	1.49	9	16
Gender	0.50	0.50	0	1
Family violence	3.45	2.20	0	8
Violent peers	0.64	1.18	0	8
Normative beliefs approving violence	1.80	0.55	1	4
Negative emotions	2.07	0.73	1	5
Adolescent violence	0.34	0.90	0	8

### Mediational Analysis

#### Total Effect – Adolescent Violence as the Response Variable

To investigate whether the relationship between family violence and adolescent violence is mediated by violent peer association, normative beliefs about violence, and negative emotions, a series of negative binomial regression models and linear regression models, including one base model and four mediating models, were constructed. Table 2 depicted the results of the five different negative binomial models. Model 1 in Table 2 addressed hypothesis 1. It indicated that family violence was significantly and positively associated with adolescent violence. Specifically, the regression coefficient of.13 in Model 1 represented the total relationship between family violence and adolescent violence after controlling for gender and age.

**TABLE 2:** Results of mediation effect.

Variables	Model 1	Model 2	Model 3	Model 4	Model 5
					
	DV = Adolescent violence	DV = Violent peers	DV = Normative beliefs	DV = Negative emotions	DV = Adolescent violence
					
	Regression coefficients (SE)	Regression coefficients (SE)	Regression coefficients (SE)	Regression coefficients (SE)	Regression coefficients (SE)
**Independent variable**					
Family violence	0.13 (0.03)***	0.09 (0.02)***	0.02 (0.01)***	0.07 (0.01)***	0.05 (0.03)^+^
**Mediating variables**					
Violent peers			0.07 (0.01)***	0.09 (0.02)***	0.22 (0.05)***
Normative beliefs					1.01 (.13)***
Negative emotions					.42 (.09)***
**Control variables**					
Gender	−1.27 (0.15)***	−0.29 (0.07)***	−0.17 (0.03)***	.10 (0.04)*	−1.03 (0.15)***
Age	−0.02 (0.05)	−0.04 (0.02)	−0.03 (0.01)*	0.01 (0.01)	0.02 (0.05)
Constant	−0.85 (0.71)	1.02 (0.33)**	2.12 (0.15)***	1.72 (0.20)***	−4.41 (0.77)***
Observations	1,137	1,137	1,137	1,137	1,137
Log likelihood	−785.38	−4,597.89	−4,597.89	−4,597.89	−4,597.89

^+^*p* < 0.1. *p < 0.05; **p < 0.01; ***p < 0.001.

#### Indirect Effects—Violent Peer Association, Normative Beliefs, and Negative Emotions as Response Variables

To explore the hypothesis 2 and 6, Model 3 tested how family violence and violent peer association were related to normative beliefs among the Chinese adolescents. The results from Model 3 indicated that both family violence and association with violent peers significantly predicted adolescents’ normative beliefs approving violence (*b* = 0.02, *p* < 0.001; *b* = 0.07, *p* < 0.001). Furthermore, normative beliefs toward violence were significantly and positively related to adolescent violence as shown in Model 5 (*b* = 1.01, *p* < 0.001). Taken together, the results in model 3 and model 5 support hypotheses 2 and 6.

The findings from Model 4 and Model 5 provided empirical tests of hypotheses 3 and 5. Model 4 tested how family violence and violent peer association were related to negative emotions among the Chinese adolescents. The results from Model 4 indicated that increases in family violence (*b* = 0.07, *p* < 0.001) and violent peer association (*b* = 0.09, *p* < 0.001) were positively and significantly related to the adolescent negative emotions. Moreover, Model 5 supported the hypothesis that negative emotions were positively related to adolescent violence (*b* = 0.42, *p* < 0.001).

To test the mediating effect of violent peer association on the relationship between family violence and adolescent violence, violent peer association was entered as the dependent variable in Model 2. The results of this model showed that family violence was a highly significant and positive predictor of violent peer association. This finding, in conjunction with the result from Model 5 that showed violent peer association (*b* = 0.22, *p* < 0.001) was significantly related to adolescent violence, supported hypothesis 4.

**FIGURE 2:**
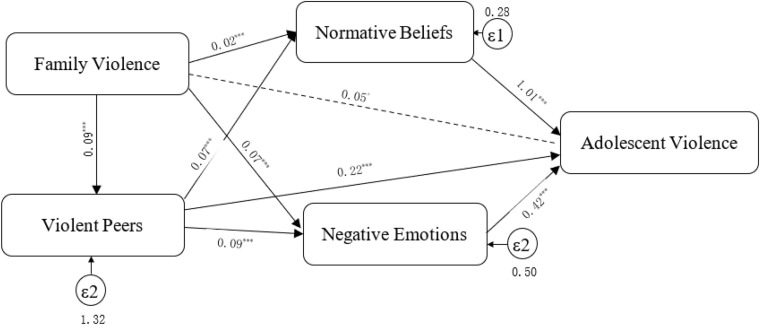
Mediation model.

All of the direct and indirect effects are shown in Figure 2. The direct effect of family violence on adolescent violence was marginally significant (*b* = 0.05, *p* < 0.10), which was shown by the dotted line in Figure 2. In contrast, the indirect effects were all significant. Specifically, all mediating variables—violent peer association, normative beliefs, and negative emotions—were significantly related to family violence and adolescent violence. As illustrated in Figure 2, much of the relationship between family violence and adolescent violence was indirect through the three mediating variables (solid lines in Figure 2).

Further decomposition of the mediation effects linking family violence to adolescent violence was shown in Table 3. The direct effect can be found in Model 5 of Table 2. An indirect effect was a product of two components. The first component was the effect of family violence on the mediator, namely violent peer association, normative beliefs, or negative emotions. The second component was the effect of the mediator on adolescent violence. As shown in Table 3, the mediating effects of violent peer association (*b* = 0.019, *p* < 0.001), normative beliefs (*b* = 0.024, *p* < 0.001), and negative emotions (*b* = 0.024, *p* < 0.01) were all statistically significant at the level of 0.01. A comparison of the direct effect and indirect effects showed that the indirect effects explained 56.8% of the overall relationship of family violence to adolescent violence. The direct effect was marginally significant (*b* = 0.054, *p* < 0.1), while the indirect effects through the three mediators were all highly significant. These results provide further evidence about the important roles of the mediators in the relationship between family violence and adolescent violence.

**TABLE 3:** Indirect Effect of family violence on adolescent violence.

Outcome	B	SE	*P*	CI	% Explained
**Direct**					
FV→VIO	0.054	0.031	0.079	−0.006 – 0.115	43.2
**Indirect**					
FV→VP→VIO	0.019	0.006	0.000	0.009 – 0.030	15.2
FV→NE→VIO	0.028	0.007	0.000	0.014 – 0.042	22.4
FV→NB→VIO	0.024	0.008	0.003	0.008 – 0.040	19.2
**Total**					
FV→VIO	0.125	0.032	0.000	0.062 – 0.189	100.0

*FV, family violence; VP, violent peers; NE, negative emotions; NB, normative beliefs; VIO, adolescent violence.*

It is also worth noting that gender consistently had a significant effect on the dependent variable in all five models in Table 2. These results indicated that gender might moderate the influences of family violence and the mediators on adolescent violence. To be specific, in the total effect, gender had a negative effect on adolescent violence (*b* = −1.27, *p* < 0.001), suggesting that female respondents had a lower level of involvement in violence than males. In the indirect effects, girls were less likely to accept the use of violence (*b* = −0.17, *p* < 0.001) and associate with violent peers (*b* = −0.29, *p* < 0.001), but more likely to suffer from negative emotions (*b* = 0.10, *p* < 0.05) than boys. The role of gender in moderating the complex relationship between family violence and adolescent violence should be further investigated in future research.

## Discussion

The current study focuses on the relationship between family violence and adolescent violence through the mediating effects of violent peer association, normative beliefs about violence, and negative emotions among Chinese adolescents. The results confirmed our hypotheses that exposure to family violence would increase adolescent involvement in violent behavior by fostering violent peer association, normative beliefs approving violence, and negative emotions. Specifically, family violence was significantly and positively related to violent peer association, normative beliefs approving violence, and negative emotions, which in turn led to more involvement in violent behavior among adolescents. The mediating effects of these variables remained significant after controlling for the influences of gender and age. In contrast, family violence had only a small and marginally significant direct effect on adolescent violence. In addition, association with violent peers was also positively related to violent beliefs and negative emotions among adolescents.

While previous studies have shown that family violence is positively associated with adolescent violence, they have not examined all the critical links connecting the two forms of violence because of the lack of attention to the roles of the mediating variables (Lichter and McCloskey, 2004; Vandivere et al., 2017). We extended this body of literature by empirically assessing the roles of several key social and psychological factors that mediated the relationship. Our findings of multiple pathways suggest that it is important to consider a full spectrum of the mediating mechanisms linking family violence to adolescent violence in order to understand the intergenerational transmission of violent behavior. However, despite our effort, there might still be other important moderators or mediators omitted from our model due to lack of data measuring those concepts. For example, prior studies have indicated that the life history strategies (Figueredo et al., 2018), empathy (Hunter et al., 2007; Li et al., 2020), and substance abuse (Beckmann et al., 2017) could either strengthen or weaken the relationship between family violence and adolescent violence. Future research can gain a more comprehensive understanding of the mechanisms contributing to the intergenerational transmission of violence by providing additional attention to the processes involving these factors.

Overall, the findings are consistent with the views of the social learning theory and the general strain theory that family violence is indirectly linked to adolescent violence through negative emotions, normative beliefs, and violent peer association (Akers, 1998; Agnew, 2013). In family environments characterized by interparental violence and physical abuse, children may emulate the confrontational patterns of interactions exhibited by family members especially their parents, which would directly increase their use of violence to resolve interpersonal conflicts. However, our analysis demonstrated that this direct effect was weak and only marginally significant. Much of relationship between family violence and adolescent violence was indirect through the mediators examined in the current study. As predicted by the social learning theory, adolescents frequently exposed to violent interactions at home were more likely to approve the use of violence and to subject themselves to the influences of delinquent peers, which increased their involvement in violent behavior. Moreover, consistent with the general strain theory, our analysis showed that experiencing family violence aggravated adolescents’ emotional problems, which further precipitated their involvement in violent behavior.

Of particular note is our finding that violent peer association mediated the influence of family violence on negative emotions, normative beliefs approving violence, and ultimately adolescent violence. Delinquent peer association has been identified as the strongest predictor of juvenile delinquency in many studies (Hawkins et al., 1992; Henry et al., 2001). The critical role that violent peer association played in mediating the relationship between family violence and adolescent violence was also evident in the current study. As demonstrated in our analysis, violent peer association mediated the relationship between family violence and adolescent violence both independently and jointly through its links with negative emotions and normative beliefs about violence. Hence, while it is important to recognize the roles of normative beliefs and negative emotions in connecting family violence and adolescent violence, researchers should pay particular attention to the mediating mechanisms involving violent peer association in order to fully understand intergenerational transmission of violence from parents to children.

Among the demographic variables controlled in this study, gender emerged as a significant predictor of adolescent violence and the factors mediating the relationship between family violence and adolescent violence, including violent peer association, negative emotions, and normative beliefs approving violence. As mentioned before, male adolescents in general were more violent than female adolescents. They were also more likely to approve the use of violence and make friends with violent peers, although they were less likely to suffer from negative emotions caused by family violence. Collectively, the indirect effects from family violence to adolescent violence through violent peer association and normal beliefs were much stronger than the indirect effect through negative emotions. Hence, male adolescents appeared to be at a higher risk for violent behavior than female adolescents under the influence of family violence.

Like previous research, this study also has some limitations. First, our measures of family violence were based on the adolescent’s perceptions using self-report data. Although children’s perceptions provide a reasonable way to measure the nature and quality of parental relationship (Palmer and Hollin, 2001; Gibbs et al., 2003), it would be unreasonable to assume that they could do it without measurement error. Second, the sample employed in this study consisted of adolescents who had both a father (or father figure) and a mother (or mother figure) in the household. As such, the results of the study might not be applicable to children living in single-parent homes. Third, because the sample was drawn from only one urban area, it might not be representative of all adolescents in China. Future studies should be conducted in different regions with samples selected from different population groups to validate the observed findings of the present study.

Despite the limitations, this study brings useful insight into the development of effective intervention and prevention programs aimed at reducing violent behavior of adolescents exposed to family violence. The key is to address the risk factors linking family violence to adolescent violence, including violent peer association, normative beliefs approving violence, and negative emotions. First, school-based and community-based programs focusing on positive relationship building are needed to help these at-risk adolescents avoid delinquent association that can negatively influence their development in many different ways. After entering puberty, adolescents begin to have a stronger need for peer acceptance and thus are more susceptible to the influences of friends and acquaintances. At this stage, programs designed to facilitate positive social networking would increase the adolescents’ association with prosocial peers, which would reduce their likelihood of developing antisocial beliefs and emotional problems that are conducive to delinquent and violent behavior (Guerra et al., 2003). Moreover, cognitive and behavioral programs can be implemented to help adolescents impacted by family violence understand the potential harms of violent interactions, strengthen their abilities to recognize and reject violent norms, and lean to use nonviolent means to resolve interpersonal conflicts. Furthermore, emotion regulation programs can be arranged for children to learn how to deal with the negative emotions caused by violence at home. By improving adolescents’ ability to regulate negative emotions, these programs can relieve the pain and stress resulted from experiencing family violence, which would reduce the possibility of perpetrating violence. Moreover, in light of our finding that interparental violence and child physical abuse might facilitate adolescent violence, it would be useful to implement parenting education programs to increase parents’ awareness of the adverse impact of family violence on adolescent development for the prevention of adolescent violence. Lastly, considering that male adolescents are more heavily involved in violence and more likely to be impacted by family violence than female adolescents, strategists and service providers should pay special attention to male adolescents who have been exposed to high levels of family violence and help them reduce their risks of future violence.

## Data Availability Statement

The dataset used in this article is not publicly available because we promised the participants that their privacy and confidentiality would be strictly protected. Requests to access the dataset should be directed to ML, liangminahu@gmail.com.

## Ethics Statement

The studies involving human participants were reviewed and approved by Research Services and Knowledge Transfer Office, University of Macau. Written informed consent to participate in this study was provided by the participants’ legal guardian/next of kin.

## Author Contributions

SL contributed to the conceptualization, funding acquisition, project administration, and supervision of the study. RX and ML contributed to the formal analysis. ML, XZ, and WT contributed to the investigation. ML contributed to the study methodology. XZ and WT contributed to the resources. RX contributed to the validation. RX, ML, XZ, and WT contributed to the writing—original draft. SL, RX, ML, XZ, and WT contributed to the writing—review and editing. All authors have read and agreed to the published version of the manuscript.

## Conflict of Interest

The authors declare that the research was conducted in the absence of any commercial or financial relationships that could be construed as a potential conflict of interest.

## Appendix 1

**TABLE TA1:** Measurement of negative emotions.

Subscale	Item
Anxiety	1. I feel nervous or can easily become nervous.
	2. I feel restless.
	3. I feel scared for no reason.
	4. I feel upset.
	5. I feel insecure inside.
	6. I always feel something is not right.
Depression	7. I feel depressed.
	8. I can easily cry.
	9. I feel no hope toward the future.
	10. I often blame myself.
	11. I often feel listless and lack of energy.
	12. I often have suicide thought.
Hostility	13. I often lose my temper and have trouble controlling myself.
	14. Yelling or throwing something.
	15. I often argue with others.
	16. I get excited and upset easily.
	17. I have the impulse to hut or hit others.
	18. I have the impulse to break things.
Paranoid ideation	19. I always think differently than others.
	20. I think others want to take advantage of me.
	21. I always feel that someone is talking about me behind my back.
	22. I think most people cannot be trusted.
	23. Other people’s evaluation of my performance is inappropriate.
	24. I always feel that others are against me.
Interpersonal sensitivity and strain	25. I feel that others are unfriendly to me, and do not like me.
	26. I feel uncomfortable when others are looking at me or talking about me.
	27. I feel that others don’t understand me, and have no sympathy for me.
	28. My feelings are easily hurt by others.
	29. I feel shy and uncomfortable when I am with the opposite sex.
	30. I want others to be perfect.
